# Carcinoma

**Published:** 1906-05-19

**Authors:** 


### Progress in Medicine and Surgery.

### CARCINOMA.

**On the Method of the Spread and Dissemination of Carcinoma and its Influence on Treatment.**—Whilst we vainly wait for any definite results from the many researches into the cause and prevention of cancer, the subject of its radical removal must remain that of the most practical importance in the study of the disease. It has been generally held that carcinoma is spread by a process of embolism of the cancer-cells in the lymphatic and blood-vessels. But in the case of the breast, Handley [1] has shown that this view must be modified. According to this authority the cancer-cells actually spread along the lymphatic channels by a process of continuous growth, and that this permeating growth takes place as readily in a direction contrary to the lymph stream as with it. The first difficulty in accepting this theory is the fact that if a late case of mammary carcinoma be examined, the region immediately round the primary growth contains no permeated cancerous lymphatic trunks. Isolated nodules of growth are present which suggest the position of emboli. But it appears that if an area of the tissues at a greater distance from the growth be examined microscopically, it will be found that the lymphatic trunks are all permeated with cancer-cells. Thus, in every case three distinct zones may be distinguished. First, in the centre is the primary growth; round this is a zone in which the lymphatics are devoid of cancer-cells, but in which isolated nodules of growth may be present; and a third zone, which appears normal to the naked eye, forms the true margin of the growth, and here the lymphatic channels are filled with malignant cells. This last zone may be described as the microscopic edge of the growth. To explain the freedom of the lymph channels from cancer-cells in the intermediate zone, it is stated that a process of peri-lymphatic leucocytosis is set up by the malignant cells growing in the lymph vessels, and that this causes a fibrosis which destroys them. So that a double process of peripheral extension and central destruction of the carcinoma proceed at the same time, and the whole method of growth may be compared to that of a ringworm or serpiginous ulcer. But at the same time that the cancer-cells are permeating the lymphatic vessel, they may be forced into the lymph capillaries outwards to the skin or inwards to the body wall. And when in these situations the cancer-cells break through the walls of the lymph space, they then form the nucleus of a cancer nodule. In confirmation of this theory of the centrifugal spread of carcinoma are the facts of the distribution of the secondary malignant nodules in the skin and of the deposits in bones. The nodules in the skin always make their first appearance close to the primary growth, and later at points successively more and more remote from it. The area within which they are found is invariably a circle of continually increasing diameter with the primary growth at its centre. And in those rare cases when nearly the whole trunk is covered by secondary nodules, the arms below the deltoid insertion, and the legs below the middle of the thighs, always remain free. In bone metastases, too, as seen in 329 cases observed during thirty years at the Middlesex Hospital, those bones are most frequently affected which lie nearest to the primary growth. Thus, the sternum, ribs, femur, spine, humerus are the bones most often invaded, and that in the order mentioned. The fact that the femur and humerus are affected more often than such bones as the scapula is explained by the intimate relationship which the deep fascia has to the former in the situation of the trochanter and the deltoid insertion. But no explanation is offered of the facts that the clavicle is seldom affected, less often indeed, than the cranial bones, or that the bodies of the vertebræ are involved rather than the spines and laminæ. But only one case in this whole series shows involvement of bones distal to the knee or elbow, and therefore it is impossible to regard bony metastases at any rate, as embolic in origin, because if this were so it would be the distal bones which would be most often the seat of the growths. As regards visceral invasion, the same method of direct permeation of lymph channels is said to take place. In this there are three stages. First the network of lymph vessels which lies in the deep fascia is involved, then the deep branches of these vessels which perforate the body wall, and lastly the serous membrane which lines the thorax and abdomen, carries the cancerous process to the viscera. If the blood were the main channel of diffusion the thoracic viscera would naturally be invaded more frequently than the abdominal. But this is not the case.[2] Indeed, the abdominal viscera are more often attacked than the thoracic, and in 12 per cent. of autopsies the abdominal viscera present metastatic growths, while the thorax is quite free. This is explained on the permeation hypothesis by the fact that in the linea alba there exists a weak spot in the defence of the body cavity where the cancer-cells find a short path from the fascial lymphatics to those of the abdominal cavity. The facts brought forward are so clear that the truth of these propositions must be admitted. But at the same time it is difficult to understand why general peritoneal infection by carcinoma is not more common than isolated deposits in the viscera. But of the importance of the deep fascia in the process of disseminating cancer there can be no doubt, and it is also probable that the skin plays quite a secondary part, as it contains only terminal lymph capillaries, and not the main lymph vessels. From this it follows that in the radical operation for mammary cancer more attention must be paid to a wide removal of deep fascia, especially in the epigastric region, and less attention need be paid to a wide removal of skin. Thus, a circular incision about five inches in diameter will suffice to remove the primary growth, together with the lymphatic glands, and then by radiating incisions upwards and downwards the skin can be turned up and the deep fascia removed as far as the mid-line and down to (and including) that covering the epigastrium. The skin can then be easily brought together without the necessity of skin grafting. If after this secondary nodules appear on the skin, the course to be pursued depends upon their position. If they overlie the area from which the deep fascia has been removed, they may be regarded merely as isolated growths and removed by a small incision. But if they overlie parts in which the deep fascia has been left, a widespread area of underlying permeated lymphatics is indicated, and operation will be useless. Cheatle[3] discusses the same problems of the spread of cancer in the case of epithelioma of the tongue. He bases his observations on sixteen cases of advanced disease which were removed by operation. It is a peculiar fact that the disease is often limited by the mid-line, and also that it seldom spreads from behind to the tip of the tongue. But this limitation of the growth by the mid-line in advanced cases is only superficial, and the cancer has often invaded the opposite side deep in the substance of the organ when in the surface it seems to be unilateral. This shows that the median raphé has nothing to do with the limitation of growth, because it is well marked in the depth of the tongue and is not present in its surface. In all the advanced cases the extrinsic muscles were involved in the growth, and, indeed, it seems that it is along the actual muscle fibres that the growth extends, rather than, as might have been thought, in the inter-muscular septa. The hyoglossus, the genioglossal portion of the genio-hyoglossus, and the inferior lingualis muscles are specially liable to involvement. It is therefore better in all advanced cases to attempt to remove these muscles right down to their attachment to the hyoid bone.

[1] Glasgow Med. Jour., Dec. 1905. [2] Med. Rec., Sept. 2, 1905. [3] Pract., Sept. 1905.

(*To be continued.*)

